# Supplementary material for: Transcriptome Analysis Reveals Vimentin-Induced Disruption of Cell–Cell Associations Augments Breast Cancer Cell Migration
Source: Cells. 2022 Dec 13;11(24):4035. doi: 10.3390/cells11244035 (PMC9776984; doi:10.3390/cells11244035)
Supplement: Supplementary file 1 [file cells-11-04035-s001.zip › DEGs and enrichment analysis.zip!/DEGs and enrichment analysis/WT_VIMvsVector GO enrichment/DOWN/WT_VIMvsVector_down.GObp_DAG.pdf]

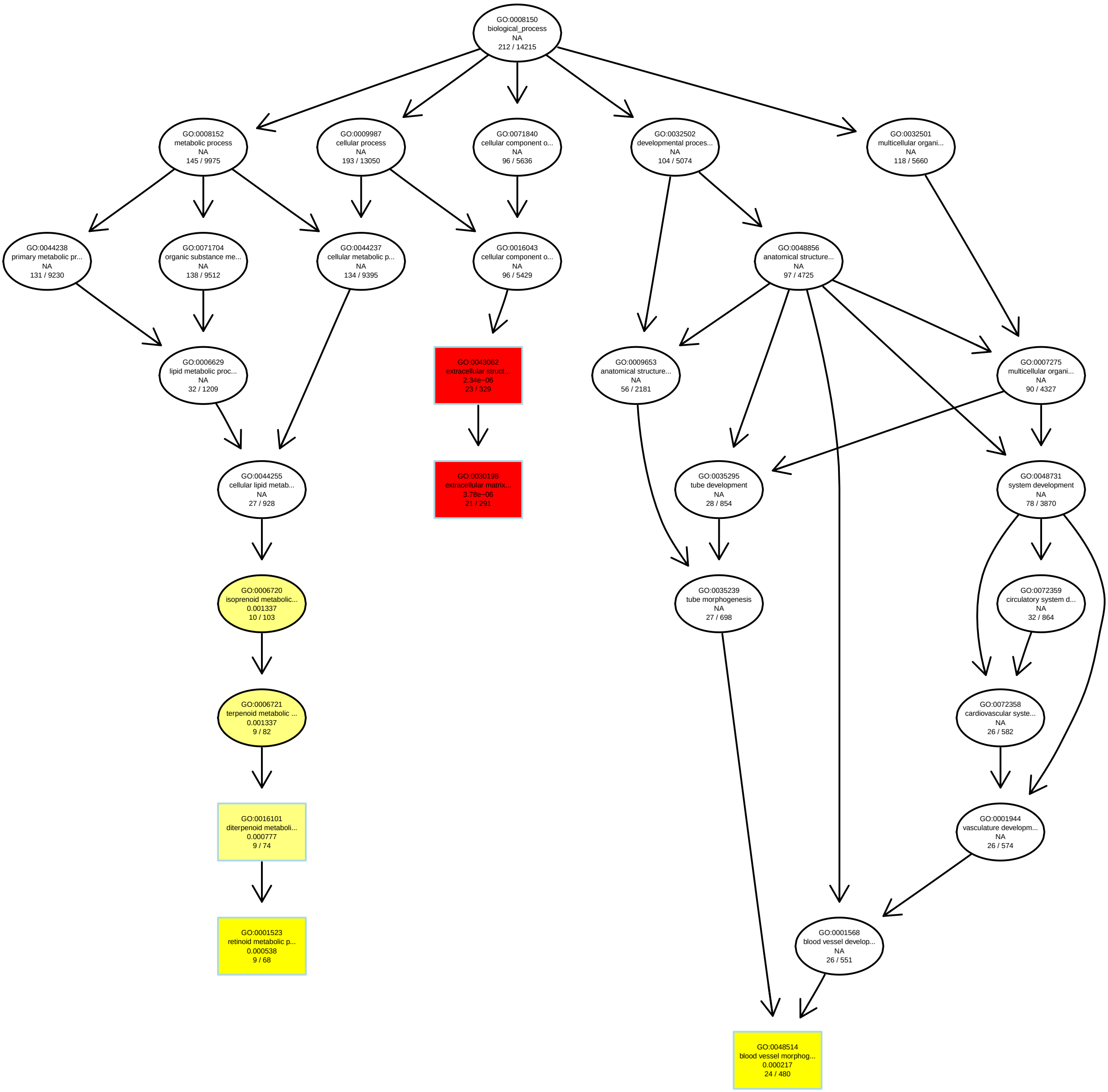
GO:0008150
biological_process
NA
212 / 14215
GO:0008152
metabolic process
NA
145 / 9975
GO:0009987
cellular process
NA
193 / 13050
GO:0071840
cellular component o...
NA
96 / 5636
GO:0032502
developmental proces...
NA
104 / 5074
GO:0032501
multicellular organi...
NA
118 / 5660
GO:0044238
primary metabolic pr...
NA
131 / 9230
GO:0071704
organic substance me...
NA
138 / 9512
GO:0044237
cellular metabolic p...
NA
134 / 9395
GO:0016043
cellular component o...
NA
96 / 5429
GO:0048856
anatomical structure...
NA
97 / 4725
GO:0006629
lipid metabolic proc...
NA
32 / 1209
GO:0043062
extracellular struct...
2.34e-06
23 / 329
GO:0009653
anatomical structure...
NA
56 / 2181
GO:0007275
multicellular organi...
NA
90 / 4327
GO:0044255
cellular lipid metab...
NA
27 / 928
GO:0030198
extracellular matrix...
3.78e-06
21 / 291
GO:0035295
tube development
NA
28 / 854
GO:0048731
system development
NA
78 / 3870
GO:0006720
isoprenoid metabolic...
0.001337
10 / 103
GO:0035239
tube morphogenesis
NA
27 / 698
GO:0072359
circulatory system d...
NA
32 / 864
GO:0006721
terpenoid metabolic ...
0.001337
9 / 82
GO:0072358
cardiovascular syste...
NA
26 / 582
GO:0016101
diterpenoid metaboli...
0.000777
9 / 74
GO:0001944
vasculature developm...
NA
26 / 574
GO:0001523
retinoid metabolic p...
0.000538
9 / 68
GO:0001568
blood vessel develop...
NA
26 / 551
GO:0048514
blood vessel morphog...
0.000217
24 / 480
